# The Hunt for the Source of Primary Interleukin-4: How We Discovered That Natural Killer T Cells and Basophils Determine T Helper Type 2 Cell Differentiation *In Vivo*

**DOI:** 10.3389/fimmu.2018.00716

**Published:** 2018-04-23

**Authors:** Tomohiro Yoshimoto

**Affiliations:** ^1^Department of Immunology, Hyogo College of Medicine, Nishinomiya, Japan; ^2^Laboratory of Allergic Diseases, Institute for Advanced Medical Sciences, Hyogo College of Medicine, Nishinomiya, Japan

**Keywords:** Dr. William E. Paul, T helper type 2 cell differentiation, interleukin-4, interleukin-18, natural killer T cell

## Abstract

Interleukin (IL)-4 plays a central role in determining the phenotype of naïve CD4^+^ T cells by promoting their differentiation into IL-4-producing T helper type 2 (Th2) cells, which are crucial for the induction of allergic inflammation. However, to date, the potential sources of “primary IL-4” *in vivo*, as distinguished from IL-4 produced by Th2 cells, remain unclear. Here, I describe the research I carried out in collaboration with Dr. William E. Paul to identify “primary IL-4”-producing cells and Th2 cell differentiation *in vivo*.

## Introduction

In 1986, Coffman and Mosmann proposed the T helper (Th) dichotomy, in which they showed the presence of two different cell subsets, consisting of Th1 and Th2 CD4^+^ T cell lineages each expressing a definite cytokine profile (1). CD4^+^ T cells are differentiated into Th1 cells in the presence of interleukin (IL)-12, which primarily produce IFN-γ and IL-2 and are concerned in cell-mediated immune responses. IFN-γ activates macrophages and is extremely efficient in the elimination of intracellular pathogens. While CD4^+^ T cells are differentiated into Th2 cells in the presence of IL-4 and produce IL-4, IL-5, IL-9, and IL-13 (2, 3), these Th2 cytokines are critical for the development of allergic diseases and the elimination of helminth infections by the induction of IgE synthesis, the activation of basophils and mast cells, and the recruitment of eosinophils. The theory of a Th1/Th2 balance presented the base for understanding the mechanisms of immune responses and has been generally established as a paradigm of the immune system for over 30 years.

**Figure 1:**
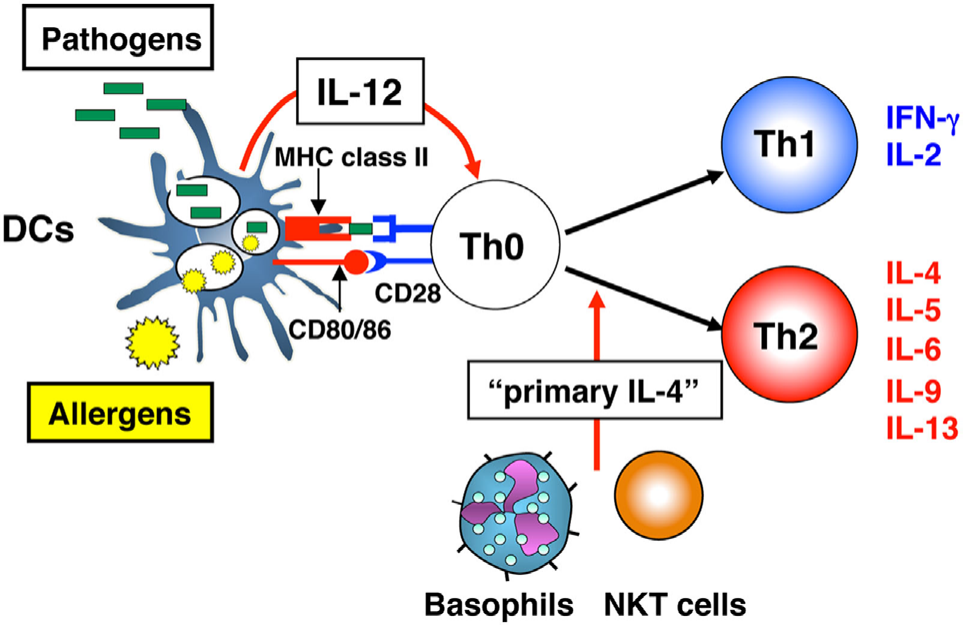
Differentiation of naïve CD4^+^ T cells into T helper (Th)1 or Th2 cells. Differentiation of naïve CD4^+^ T cells into Th1 or Th2 cells requires three signals: (1) T cell receptor (TCR) triggering through peptide-antigen recognition in the context of MHC class II molecules; (2) augmentation of TCR signaling *via* CD80 and/or CD86 and CD28 co-stimulatory molecules; and (3) an appropriate cytokine, interleukin (IL)-12 for Th1 cell differentiation and IL-4 for Th2 cell differentiation. For Th1 cell differentiation, which develops in response to viral and bacterial pathogens, dendritic cells (DCs) function as antigen-presenting cells and provide all three signals. For Th2 cell differentiation, which develops in response to an allergen, DCs cannot provide all three required signals, because of the lack of “primary IL-4,” the cytokine essential for Th2 cell differentiation. Cells, such as natural killer T (NKT) cells or basophils are candidate “primary IL-4”-producing cells.

It is well established that the differentiation of naïve CD4^+^ T cells into Th1 or Th2 cells requires three signals: (1) T cell receptor (TCR) triggering through antigen recognition by MHC class II molecules; (2) augmentation of TCR signaling *via* co-stimulatory molecules, such as CD80 and/or CD86 and CD28; and (3) an appropriate cytokine, e.g., IL-12 for Th1 cell (4, 5) and IL-4 for Th2 cell differentiation (6, 7). In the early 1990s, the *in vivo* source of IL-12, essential for Th1 cell differentiation, was revealed as macrophages or dendritic cells (DCs) in response to pathogens (4, 8). By contrast, potential *in vivo* sources of IL-4, essential for Th2 cell differentiation, remained unclear. Therefore, Dr. Paul gave me a mission to identify sources of IL-4 that promote the differentiation of naïve CD4^+^ T cells into IL-4 producers. This type of IL-4 was designated as “primary IL-4” to distinguish it from Th2 cell-producing IL-4 (Figure 1).

We first discovered a specific subpopulation of helper T cells, CD4^+^NK1.1^+^ T cells, which promptly produce significant amounts of IL-4 upon stimulation *in vivo* (9). Next, we showed the property of basophils as “primary IL-4”-producing cells (10). Finally, we revealed that basophils have dual functions as “primary IL-4”-producing cells and as antigen-presenting cells (APCs) which preferentially induce Th2 cells *in vivo* and *in vitro* (11). In this review, I describe the story of research to identify “primary IL-4”-producing cells and Th2 cell differentiation in collaboration with Dr. William E. Paul.

## CD4^+^NK1.1^+^ T Cells are a Source of IL-4 that Promotes the Differentiation of Naïve CD4^+^ T Cells into Th2 Cells

In 1994, Dr. Paul and I showed that almost all amounts of IL-4 produced within 30–90 min after an injection of antibody against anti-CD3 into mice were from an unexpected population of CD4^+^ T cells that express receptors of the NK lineage, NK1.1, on their surface (9). These CD4^+^NK1.1^+^ T cells are somewhat small in the spleen (~1% of splenic cells) and have a specific TCR expression of Vα14 and Vβ8.2, which are specific for MHC class I-like molecules CD1. Today, these cells are termed natural killer T (NKT) cells (12, 13).

Interestingly, the development of NKT cells was markedly impaired in β2-microglobulin deficient (β2M^−/−^) mice (14). This is in keeping with the association of β2-microglobulin with CD1. Indeed, splenic cells from β2M^−/−^ mice produced little or no IL-4 in response to *in vivo* treatment with anti-CD3 antibody (15). Furthermore, β2M^−/−^ mice impaired the presence of IL-4-producing cells 5 days after an injection of goat anti-mouse IgD antibody and produced minimal or no IgE in response to this stimulation. Furthermore, the ability of irradiated β2M^−/−^ mice to produce IgE in response to an *in vivo* challenge with anti-IgD antibody can be restored by transferring purified populations of CD4^+^NK1.1^+^ thymocytes and T cell-depleted splenic cells from normal mice (15). These results show that the production of IgE depends upon NKT cells, probably because NKT cells can rapidly produce “primary IL-4,” which sequentially prime naïve CD4^+^ T cells to differentiate into IL-4-producing Th2 cells.

SJL mice have a defect in IgE production to a variety of stimulants (16, 17). To reveal the possibility that their defect might be due to a lack of splenic NKT cells, SJL mice were *in vivo* challenged with anti-IgD antibody. As a result, SJL mice had defects in IgE production and IL-4-producing cells in response to this treatment. By contrast, similarly, anti-IgD-treated BALB/c and C57BL/6 mice made substantial amounts of IgE and induced IL-4-producing Th2 cells. In addition, *in vivo* treatment of SJL mice with anti-CD3 antibody also failed to produce “primary IL-4” (18).

These results suggest that the defect in IL-4 and IgE production in two strains of mice—β2M^−/−^ mice and SJL mice—was associated with, and might be caused by, an absence of the NKT cells. However, we observed that in response to certain stimulant, β2M^−/−^ mice produced IgE. These mice immunized with ovalbumin (OVA) and alum-induced IgE production and IL-4-producing cells (TY and WEP, unpublished work). This may be explained by the production of “primary IL-4” by cell types other than NKT cells.

When Dr. Paul and I published these attractive data, we considered several possibilities when answering the question, “How do NKT cells contribute to Th2 cell differentiation *in vivo*?” as described below. First, peptides derived from allergens or Th2-inducing pathogens, such as helminths, may connect to CD1 molecule and form epitopes recognized by NKT cell receptors. The second possibility is that APCs that interrelate with allergens or Th2-inducing pathogens may regulate the expression level of CD1 or co-stimulatory molecules on their surface. The third possibility is that NKT cells may receive a robust stimulus through the interaction of their receptors with CD1 expressed on the organs such as skin, respiratory tract, and gut. If naïve CD4^+^ T cells encounter antigens in these organs, they are initiated by “primary IL-4” and differentiated into Th2 cells.

## NKT Cells Respond to IL-18 to Produce IL-4 that Promotes Naïve CD4^+^ T Cells to Differentiate into Th2 Cells

### IL-18 Induction of IgE: Dependence on CD4^+^ T Cells and IL-4

In 1995, when I returned to Japan, a new cytokine IL-18 was discovered and cloned at Hyogo College of Medicine (19). IL-18, an IL-1-like cytokine that requires cleavage by caspase-1 to become active form, was originally recognized as a factor that enhanced IFN-γ production by Th1 cells in the presence of antigen plus IL-12 (19, 20). However, our later studies and those of others revealed that without IL-12 stimulation, IL-18 promotes Th2 cytokine production by CD4^+^ T cells, basophils, and mast cells (21–25). With IL-3, IL-18 stimulates basophils and mast cells to induce IL-4, IL-9, and IL-13 production even without cross-linkage of FcεRI (21). Naïve CD4^+^ T cells cultured with IL-2 and IL-18 without engagement of TCR for 4 days produced moderate and significant amounts of IL-4 and IL-13, respectively (23). Additional stimulation with antibodies against CD3 and CD28 increased their capacity to produce IL-4 and IL-13. Moreover, these activated T cells were differentiated into Th2 cells *in vitro*, while naïve CD4^+^ T cells cultured with the same protocol, but with additional neutralizing antibody to IL-4, were differentiated into Th1 cells, not Th2 cells. These results suggested that IL-18 has the potential to develop Th2 cells in an IL-4-dependent manner (23).

We also demonstrated that in addition to IL-4 production, naïve CD4^+^ T cells stimulated with IL-2 and IL-18 for 4 days upregulated CD40 ligand (CD40L) and induced B cells to secrete IgE *in vitro* (23). Consistent with these findings, the daily injection of IL-18 into mice induced a significant, dose-dependent increase in serum IgE levels *in vivo* in an IL-4-dependent fashion (23, 24). In addition, transgenic mice overexpressing human caspase-1 in keratinocytes, which have significant increased serum levels of mature IL-18, spontaneously develop atopic dermatitis with high serum levels of IgE. This IgE response disappeared in caspase-1 Tg mice lacking IL-18 or STAT6, a crucial intracellular element for IL-4-signaling pathway, indicating that IL-18- and IL-4-mediated signaling pathways are contributed to their IgE response (23). These results taken together indicate that IL-18 has the potential to induce Th2 cell differentiation. In these experiments, Dr. Paul and Dr. Nancy Noben-Trauth collaborated with us in the evaluation of IL-18-induced IgE response *in vivo* and showed that it was IL-4-dependent using BALB/c IL-4Rα^−/−^ mice (23).

### IL-18-Stimulated NKT Cells Are the Major Source of IL-4

**Figure 2:**
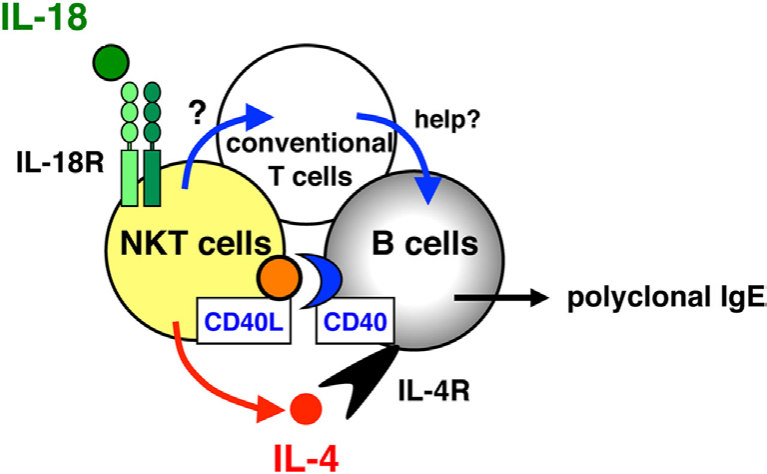
Role of natural killer T (NKT) cells and conventional T cells for interleukin (IL)-18-induced IgE production *in vivo*. NKT cells, strongly positive for the IL-18Rα chain, are a critical subset of CD4^+^ T cells that respond to IL-18 by the expression of IL-4 and CD40 ligand (CD40L) *in vivo*, and conventional CD4^+^ T cells act as helper T cells together with NKT cells in IL-18-induced IgE responses.

Although it was clearly demonstrated that CD4^+^ T cells can respond to IL-18 to produce IL-4 *in vivo* and *in vitro* (23, 24), the subset of CD4^+^ T cells that responded to IL-18 stimulation *in vivo* by inducing the expression of IL-4 and CD40L remained unidentified. Collaborating with Dr. Paul and Dr. Booki Min, we revealed that NKT cells are the target cells for IL-18 as described below (26). The daily injection of IL-18 resulted in increased serum levels of IgE, IL-4, and IL-13 in normal mice but not in CD1^−/−^ mice lacking NKT cells, because NKT cells are positively selected by MHC class I-like molecules CD1 (12). In addition, compared with conventional CD4^+^ T cells, NKT cells, strongly positive for the IL-18Rα chain, produced large amounts of Th2 cytokines (IL-4, IL-9, and IL-13) and increased their CD40L expression in response to IL-18 plus IL-2 *in vitro* without TCR engagement. Moreover, IL-18- and IL-2-stimulated NKT cells induced *in vitro* IgE isotype switching in B cells. By contrast, MHC class II^−/−^ mice, which lack conventional CD4^+^ T cells but have NKT cells, failed to produce IgE in response to IL-18 treatment, indicating that conventional CD4^+^ T cells are important for IL-18-induced IgE production. Actually, these mice, reconstituted with conventional CD4^+^ T cells from wild type but not from IL-4^−/−^ mice, produced IgE. Thus, these results demonstrated that NKT cells are an essential subset of CD4^+^ T cells responding to IL-18 by inducing IL-4 production and CD40L expression *in vivo*, and IL-4-producing conventional CD4^+^ T cells are required for IgE production by B cells together with NKT cells (Figure 2).

Natural killer T cells rapidly produce IL-4 after stimulation of the TCR with anti-CD3 antibody (9). Furthermore, without TCR engagement, NKT cells produce a variety of Th2 cytokines including IL-4 in response to IL-18, which has the potential to initiate Th2 cell development and IgE production (26). Therefore, our original work with Dr. Paul on the roles of NKT cells in Th2 cell development at the National Institutes of Health in the USA (9) connected to the discovery of the contribution of NKT cells for IL-18-driven Th2 cell development in Japan (23, 26). Throughout these experiments, Dr. Paul gave us a great deal of support and engaged with us in helpful discussions.

## Recent Studies of NKT Cells as IL-4-Producing Cells

NK1.1 has been considered to be a marker of NKT cell; however, it is neither expressed in BALB/c mice. Thus, instead of surface markers, recent study performed intracellular staining for transcription factors recognized consistently in different mouse strains. According to the combination of transcription factor (T-bet, GATA-3, and ROR-γt), NKT cells are separated into three distinctive subsets: NKT1, NKT2, and NKT17 cell, analogous to the nomenclature of Th lineage (Th1/Th2/Th17) (27). Lee et al. demonstrated that NKT2 cells highly expressed Th2-specific transcription factor GATA-3, while NKT1 cells expressed a high level of T-bet with low GATA-3. Notably, upon stimulation with PMA plus ionomycin, thymic NKT1, NKT2, and NKT17 cells produced IFN-γ, IL-4, and IL-17, respectively. Compared with C57BL/6 mice, BALB/c mice have the greater abundance of NKT2 cells and secrete large amounts of IL-4 at a steady state. It is well known that C57BL/6 and BALB/c mice are the prototypes of strains dominating Th1 and Th2 responses, respectively. BALB/c mice have higher serum IgE levels than C57BL/6 mice, while BALB/c background CD1d^−/−^ mice lacking NKT cells significantly reduced serum IgE at a steady state (27). Thus, NKT2 cell-derived IL-4 might modify immune responses under normal steady-state conditions, conceivably contributing to Th2 dominance in BALB/c mice.

Very recently, it has been reported that NKT cells might represent the early source of IL-4 for the initiation of antiviral B cell immunity (28). B cells are essential for the defense against pathogenic infections through the production of pathogen-specific antibodies in germinal centers. In this process, follicular helper T (TfH) cells are known to regulate the initiation of antiviral B cell immunity *via* co-stimulatory molecules and cytokines, such as IFN-γ, IL-4, and IL-21 (29). However, the mechanism by which B cells initially seed germinal center reactions remains unclear. Gaya et al. demonstrated that during influenza infection, there are two waves of IL-4 production: an early wave, mainly produced by NKT cells and restricted to the periphery of B cell follicles, and a late wave, produced by germinal center-resident TfH cells. Furthermore, close interactions between NKT cells and resident macrophages at the follicular through CD1d are necessary to induce early IL-4 production by NKT cells by 3 days after infection. Interestingly, this early IL-4 production by NKT cells was significantly reduced in IL-18R^−/−^ mice, suggesting that IL-18 enhances IL-4 secretion by NKT cells as we reported previously (26). Indeed, they detected a strong accumulation of IL-18 in both subcapsular sinus and medullar macrophages on day 2 of influenza infection, suggesting that these resident macrophages are a source of IL-18. Therefore, early IL-4 production by IL-18-stimulated NKT cells might contribute to the initiation of antiviral B cell immunity.

## Basophils are “Primary IL-4”-Producing Cells

Before serial experiments with NKT cells for Th2 cell development *in vivo*, Dr. Paul had an idea that “primary IL-4”-producing cells might be activated T cells themselves or FcεRI^+^ cells, cells with the morphology of basophils (30, 31). However, neither of these cells appeared ideally suitable to be a physiological source of “primary IL-4” for Th2 cell differentiation. Specifically, the main problem with the theory that basophils might be “primary IL-4”-producing cells is that the only established physiological pathway through which these cells are stimulated to produce IL-4 is by the cross-linkage of FcεRI. In other words, basophil-IL-4 production is dependent upon established Th2 responses of IgE production. However, several studies revealed that basophils might be “primary IL-4”-producing cells for Th2 cell differentiation as described below.

We revealed that without FcεRI cross-linkage, IL-18 stimulated basophils and mast cells to produce Th2 cytokines (21). Murine bone-marrow-derived basophils and mast cells express IL-18Rα chain and produce Th2 cytokines (IL-4, IL-6, IL-9, and IL-13) and histamine in response to IL-3 plus IL-18 stimulation (21). In addition, murine basophils and mast cells express ST2, the receptor for IL-33, a member of the IL-1 family (10). Like IL-18, IL-33 stimulates basophils and mast cells to produce Th2 cytokines without FcεRI cross-linkage. Notably, basophils but not mast cells produce IL-4 in response to IL-3 plus IL-18 or IL-3 plus IL-33 (10).

Proteases secreted from helminths and protease allergens from house dust mites can also induce Th2 cytokines (IL-4, IL-5, and IL-13) from human basophils purified from peripheral blood. Protease inhibitors blocked the production of these Th2 cytokines, suggesting that proteolytic antigens can directly activate basophils (32). Moreover, a cysteine protease allergen papain significantly induced the expression of Th2 cytokines and TSLP in murine basophils (33). Although the receptor or sensors that recognize proteases from allergens and helminths on basophils remain unknown, the downstream signaling pathway activated by papain in basophils was recently characterized (34).

Human basophils express Toll-like receptor (TLR) 2 and produce Th2 cytokines when stimulated with several TLR2-specific ligands (35). We reported that murine basophils selectively express TLR1, 2, 4, and 6 and produce Th2 cytokines in response to IL-3 plus peptidoglycan or IL-3 plus lipopolysaccharide *via* TLR2 or TLR4, respectively (11). It is well known that some infectious conditions induce allergic inflammatory responses. Thus, pathogen-induced Th2 cytokine production from basophils *via* TLRs may contribute to the onset of allergic diseases.

Basophils produce IL-4 significantly and promptly in response to various stimuli, such as IL-18, IL-33, proteases, and TLR ligands, making them a potential candidate for the source of “primary IL-4.” Indeed, Min and colleagues reported that in the presence of DCs and antigen, basophils initiated Th2 cell differentiation *in vitro* (36). They showed that naïve CD4^+^ T cells could be differentiated into Th2 cells if they were stimulated with antigen in the presence of basophils and DCs without additional IL-4 (36). Basophil-mediated Th2 cell differentiation was mainly mediated by the IL-4 produced by basophils, because Th2 cell differentiation was not detected when IL-4-deficient basophils were used. In addition, Min and colleagues showed that, at least *in vitro*, the Th2-promoting capacity of basophils was in part due to a direct cell–cell contact with CD4^+^ T cells (36). This led to the later finding that MHC class II expressing basophils functions as APCs, as described below. Nevertheless, their studies clearly provided a proof of principle that basophils can promote Th2 cell differentiation in the presence of DCs and antigen *via* basophil-derived “primary IL-4.”

Sokol et al. revealed that basophils are crucial for Th2 cell differentiation in response to papain *in vivo* (33). The immunization of mice with papain alone induced significant Th2 responses, Th2 cytokine production in lymph nodes, and serum papain-specific IgE. Most extraordinarily, basophils quickly migrated into T cell zones of the draining lymph nodes and produced IL-4, 3 days after the *in vivo* injection of papain (33). Taken together, these results show that “primary IL-4” produced by basophils is essential for Th2 cell differentiation. In this setting, it was initially considered that DCs functioned as APCs and induced Th2 cell differentiation in collaboration with basophil-derived “primary IL-4.”

## Basophils have Dual Functions as “Primary IL-4”-Producing Cells and as APCs that Preferentially Induce Th2 Cell Differentiation

**Figure 3:**
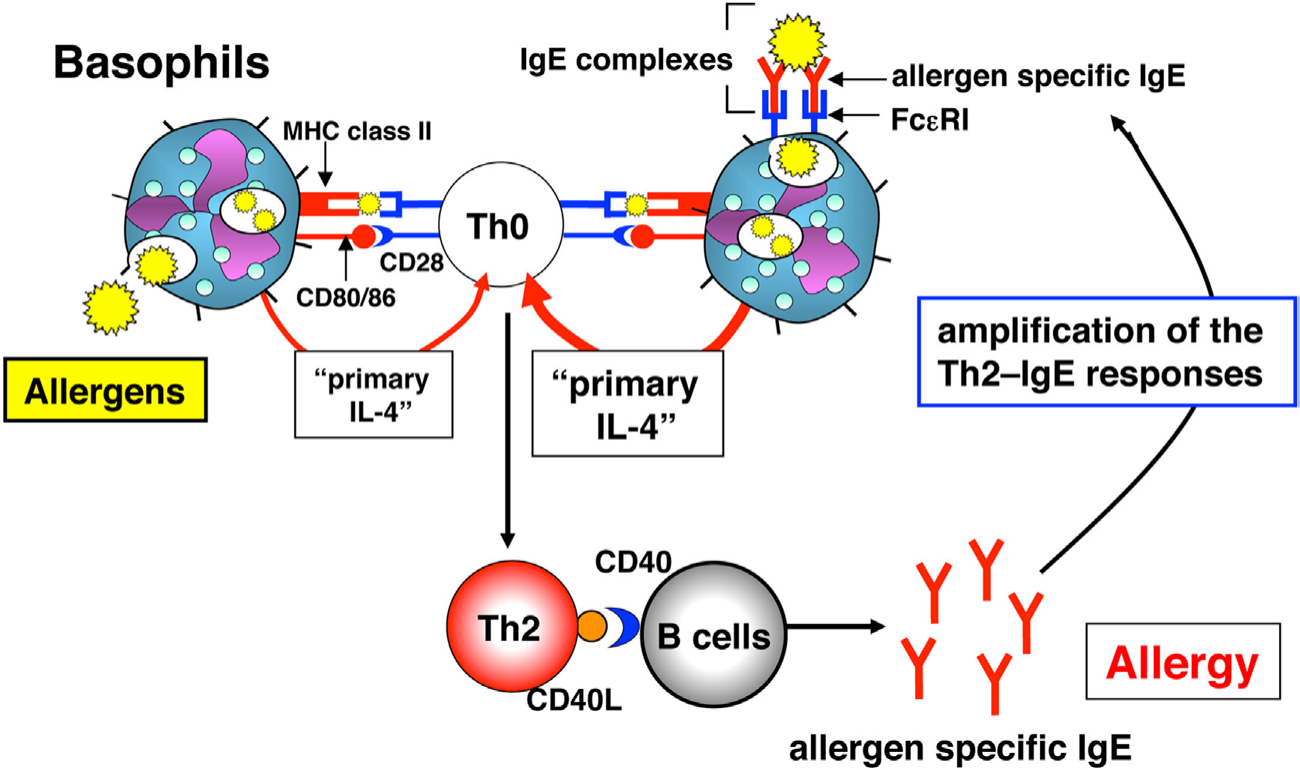
The role of basophils in the induction and amplification of T helper (Th)2 cell responses. Basophils initially induce Th2 cells *in vivo via* “primary interleukin (IL)-4” production and the presentation of complexes of allergen peptide plus MHC class II to CD4^+^ T cells. These activated Th2 cells produce IL-4 and express CD40 ligand (CD40L), which, in combination, induce B cells to proliferate and produce allergen-specific IgE *in vivo*. Immune complexes of allergen and allergen-specific IgE “preferentially” augment the development of allergen-specific Th2 responses in an endogenous basophil-dependent manner.

In 2009, three independent groups, including ours, published studies showing that besides the function of basophils as “primary IL-4”-producing cells, basophils have the function of APCs to preferentially induce Th2 cell differentiation both *in vitro* and *in vivo* (11, 37, 38). Murine basophils express MHC class II and co-stimulatory molecules (CD80 and CD86). Thus, basophils store all three characters required of Th2-promoting APCs, that is, the expression of MHC class II and co-stimulatory molecules, and the production of “primary IL-4” (Figure 3). We showed that basophils also expressed the lymph node-homing molecule CD62L, indicating their potential to migrate to lymph nodes to initiate T cell responses *in vivo* (11). Importantly, human cord blood-derived immature basophils (CD203c^+^c-Kit^−^) expressed human leukocyte antigen (HLA)-DR (~19%). Furthermore, human peripheral blood-derived mature basophils expressed HLA-DR after *in vitro* culture with IL-3 for 24 h (11). Thus, the expression of MHC class II on basophils is not specific for mouse.

Splenic basophils from gastrointestinal helminth (*Strongyloides venezuelensis*)-infected mice significantly induced naïve CD4^+^ T cells to develop into Th2 cells without exogenous IL-4. Furthermore, in the absence of DCs, bone-marrow-derived basophils strongly induced naïve OVA-specific CD4^+^ T cells to differentiate into Th2 cells *in vitro* in the presence of OVA peptide (OVA_323–339_) without additional IL-4. By contrast, splenic DCs induced Th2 cell differentiation only in the presence of IL-4. Additional IL-4 stimulation moderately increased the capability of basophils to induce Th2 cells, whereas basophils from IL-4^−/−^ mice failed to induce Th2 cells without additional IL-4 (11). From these results, we conclude that endogenous IL-4 from basophils is indispensable for the differentiation of naïve CD4^+^ T cells toward Th2 cells.

Other groups also demonstrated that basophils expressed MHC class II and promoted the MHC class II-dependent Th2 cell differentiation *in vitro* without additional IL-4 (37, 38). Sokol et al. showed that Th2 cell differentiation was increased in the presence of papain, which stimulated basophils to increase the expression of MHC class II and the production of IL-4 (37), indicating that protease allergen activated basophils to augment their presentation of allergen to CD4^+^ T cells.

Do basophils increase their potential to act as APCs when stimulated with antigen and antigen-specific IgE? Basophils pulsed with a low dose (6.2 µg/ml) or a high dose (100 µg/ml) of 2,4-dinitrophenyl (DNP)-conjugated OVA protein induced Th2 cells moderately or strongly, respectively. The addition of anti-DNP IgE to this culture, representative IgE–FcεRI cross-linkage, significantly increased Th2 cell differentiation even with a low dose (6.2 µg/ml) of OVA protein (11). These results indicated that FcεRI^+^ basophils might catch up low doses of antigen that are sufficient to augment antigen-specific Th2 cell differentiation in an IgE-dependent manner (Figure 3).

To reveal how basophils contribute to the development and the augmentation of *in vivo* Th2 cell–IgE responses, naïve mice or basophil-depleted mice were intravenously injected with the complex of DNP–OVA and anti-DNP IgE. This treatment preferentially induced OVA-specific Th2 cell differentiation in the spleens and OVA-specific serum IgG1 in naïve mice, whereas these Th2 responses were significantly diminished in basophil-depleted mice (11). These results clearly demonstrated that basophils contribute to the development and the augmentation of antigen-specific Th2 cells *in vivo* by taking up the complex of antigen and antigen-specific IgE, presenting antigen peptide along with MHC class II and producing large amounts of IL-4 (Figure 3).

## Controversies in this Field of Basophils

It was clearly demonstrated that basophils are “primary IL-4”-producing cells and that basophils have the function as APCs to promote Th2 responses both *in vitro* and *in vivo*. This paradigm shift was greeted with great enthusiasm, but also with objection (3, 39–42).

In 2009, researchers including our group used basophil-depleted mouse models based on antibody-mediated depletion strategies using FcεRIα-specific antibody (MAR-1). Some controversial evidences demonstrated that basophiles were not essential for Th2 differentiation and IgE production *in vivo* analysis using a basophil-specific deletion system. Ohnmacht et al. generated transgenic mice that express the Cre recombinase under control of regulatory elements for the mast cell protease 8 (*Mcpt8*) gene, which is expressed in basophils (43). More than 90% of basophils were constitutively deleted in *Mcpt8Cre* mice. They clearly demonstrated that papain-induced Th2 cell differentiation depended on DCs and not on basophils. Furthermore, they showed that basophils were not required for gastrointestinal helminth (*Nippostrongylus brasiliensis*)-induced type 2 immunity (43). Sawaguchi et al. established diphtheria toxin-based conditional basophil deletion mice, Bas-TRECK mice (44). OVA in alum-immunized Bas-TRECK mice showed equivalent serum OVA-specific IgE levels as control mice, indicating that basophils are dispensable for the development of a systemic IgE response (44).

Some groups have demonstrated that basophils had no functions as APCs and that DCs were essential APCs to promote Th2 responses in mouse models of inhaled house dust mite allergen (45) or helminthic infection (46). In addition, we and others demonstrated that basophils contribute to the cutaneously induced Th2 cell differentiation (47–50). Otsuka et al. reported a possible explanation for the controversial functions of basophils as APCs (48). In their model, basophils functioned as APCs and sufficiently initiated Th2 responses if the antigen was a hapten or a peptide. Other groups demonstrated the collaboration between basophils and DCs, where basophils promoted Th2 cell differentiation in combination with DCs as “primary IL-4”-producing cells (47, 49). The epicutaneous application of a vitamin D analog (49) or the subcutaneous injection of papain (47) induced the local production of TSLP in skin, which activated DCs to upregulate OX40L and migrate into the draining lymph node. Our group demonstrated that both basophils and TSLP had crucial roles in the development of cutaneously sensitized food allergy by the induction of Th2 responses (50). In that study, basophil-depleted or TSLP receptor-deficient mice were completely defective for Th2 responses against sensitized antigen. Basophils in the regional lymph nodes from mice epicutaneously sensitized with OVA produced more IL-4 than those from naïve mice. As a result, in the case of epicutaneously sensitized protein antigens, basophils were essential for the development of Th2 responses, as they are indispensable producers of “primary IL-4.” Therefore, skin is an exceptional organ where basophils have essential roles in the initiation and the development of Th2 responses. Furthermore, we demonstrated that basophil–TSLP pathways in skin were indispensable for the production of antigen-specific IgE and the development of gastrointestinal food allergy (50). Taken together, these reports demonstrated that basophils might contribute to Th2 responses in an organ-dependent manner, and skin could be a unique organ that needs basophils to induce most favorable Th2 responses. Nevertheless, the function of basophils as APCs in the development of Th2 responses is still highly controversial.

Recently, Miyake et al. revealed the functional relevance of basophils in Th2 cell differentiation (51). They demonstrated that basophils acquired complexes of peptide and MHC class II from DCs *via* trogocytosis in a cell-contact-dependent manner both *in vitro* and *in vivo*. That these peptide–MHC class II containing basophils might function as APCs and induce the differentiation of naïve CD4^+^ T cells into Th2 cells is very interesting. However, without any other APCs including DCs, we demonstrated that basophils strongly induced the differentiation of naïve OVA-specific CD4^+^ T into OVA-specific Th2 cells *in vitro* in the presence of OVA protein instead of OVA peptide (OVA_323–339_) without exogenous IL-4 (11). Thus, basophils can process OVA protein into OVA_323–339_ peptide and display peptide fragments together with MHC class II and to produce “primary IL-4.”

From the beginning of our basophil-APC experiments in 2006, Dr. Paul gave us critical suggestions and a great deal of support. After our publication (11), Dr. Paul mentioned in his review article (3) as described below “Basophils have important roles in the initiation of Th2 cell responses by producing Th2-associated cytokines in response to allergen or helminth-derived products. Basophils are also involved in the initiation of some Th2 cell responses by serving as APCs. However, the differential requirements for basophils or DCs as APCs for the induction of Th2 cell responses seem to depend on the nature of the antigens or helminths and/or the particular adjuvant used.”

## Concluding Remarks

Although IL-4 is essential for both *in vitro* and *in vivo* Th2 cell differentiation, the IL-4/IL-4R/STAT6-signaling pathway is not crucial in some instances of *in vivo* Th2 cell differentiation (52, 53). In addition to IL-4, other pathways such as GATA-3 and GATA5 (54, 55), and cytokines such as TSLP, IL-25, and IL-33 (3, 56, 57) have crucial roles in the induction of Th2 cell differentiation *in vivo*. A recent study suggested that IL-33 plays an important role in the induction and the augmentation of Th2 responses. Halim et al. demonstrated the role of group 2 innate lymphoid cells (ILC2s) in the differentiation of naïve CD4^+^ T cells into Th2 cells in the lung in response to the protease allergen papain (57). ILC2s, innate counterparts of adaptive Th2 cells, are activated by IL-33 from allergen-stimulated lung epithelial cells and produce large amounts of IL-13 (58). They showed that although IL-4 was dispensable for papain-induced Th2 cell differentiation, IL-13 derived from ILC2 was crucial since it induced the recruitment of activated CD40^+^ lung DCs into the draining lymph nodes where they promoted naïve CD4^+^ T cells to differentiate into Th2 cells (57).

In this review, I have described the long search for “primary IL-4”-producing cells and Th2 cell differentiation carried out in collaboration with Dr. Paul. We identified the key cells (NKT cells and basophils) and molecule (IL-18) involved in priming and developing *in vivo* Th2 responses. However, we will face a major challenge in trying to understand the detailed relations that shape “the nature of the immune response.”

In 1997, when we established a new Institute at the Hyogo College of Medicine in commemoration of the discovery of IL-18 (19), Dr. Paul named it the “Institute for Advanced Medical Sciences” and provided a good acronym “IAMS.” The phrase for the celebration of the initiation of the Institute: “Organizing scientists to reveal the secrets of nature for the good of man” reminds us that each of us has the responsibility to reveal “the secrets of nature for the good of man” by following Dr. Paul’s spirit.

## Author Contributions

TY performed the experiments and wrote the manuscript.

## Conflict of Interest Statement

The author declares that the research was conducted in the absence of any commercial or financial relationships that could be construed as a potential conflict of interest.
